# DCAF16-Based Covalent Handle for the Rational Design of Monovalent Degraders

**DOI:** 10.1021/acscentsci.4c00286

**Published:** 2024-05-17

**Authors:** Melissa Lim, Thang Do Cong, Lauren M. Orr, Ethan S. Toriki, Andrew C. Kile, James W. Papatzimas, Elijah Lee, Yihan Lin, Daniel K. Nomura

**Affiliations:** † Department of Chemistry, University of California, Berkeley, Berkeley, California 94720, United States; ‡ Novartis-Berkeley Translational Chemical Biology Institute, Berkeley, California 94720, United States; § Innovative Genomics Institute, Berkeley, California 94720, United States; ∥ Novartis Biomedical Research, Emeryville, California 94608, United States; ⊥ Department of Molecular and Cell Biology, University of California, Berkeley, Berkeley, California 94720, United States

## Abstract

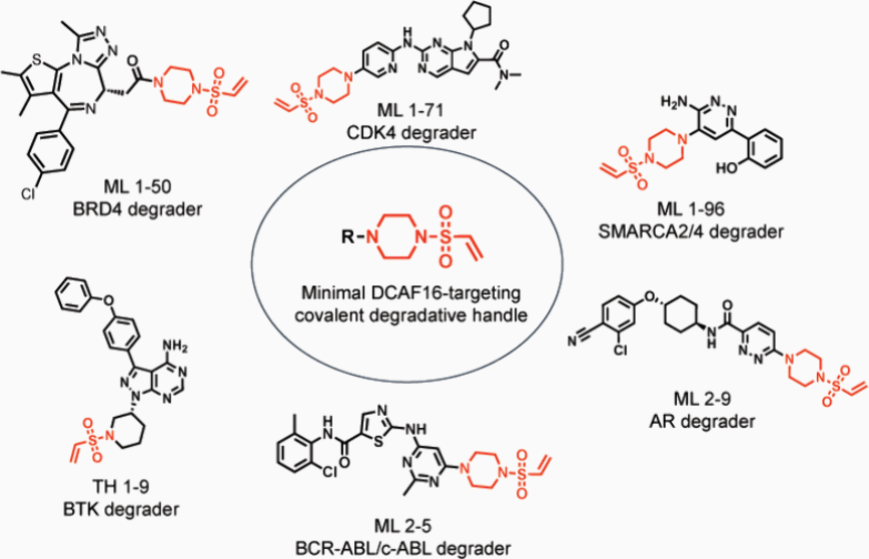

Targeted protein degradation with monovalent molecular glue degraders is a powerful therapeutic modality for eliminating disease causing proteins. However, rational design of molecular glue degraders remains challenging. In this study, we sought to identify a transplantable and linker-less covalent handle that could be appended onto the exit vector of various protein-targeting ligands to induce the degradation of their respective targets. Using the BET family inhibitor JQ1 as a testbed, we synthesized and screened a series of covalent JQ1 analogs and identified a vinylsulfonyl piperazine handle that led to the potent and selective degradation of BRD4 in cells. Through chemoproteomic profiling, we identified DCAF16 as the E3 ligase responsible for BRD4 degradation—an E3 ligase substrate receptor that has been previously covalently targeted for molecular glue-based degradation of BRD4. Interestingly, we demonstrated that this covalent handle can be transplanted across a diverse array of protein-targeting ligands spanning many different protein classes to induce the degradation of CDK4, the androgen receptor, BTK, SMARCA2/4, and BCR-ABL/c-ABL. Our study reveals a DCAF16-based covalent degradative and linker-less chemical handle that can be attached to protein-targeting ligands to induce the degradation of several different classes of protein targets.

## Abstract 2

Our study reveals a DCAF16-based covalent degradative chemical handle that can be attached to protein-targeting ligands to induce the degradation of several different classes of protein targets.

## Introduction

Monovalent molecular glue degraders have arisen as a powerful therapeutic modality for degrading therapeutic targets of interest through inducing the proximity of an E3 ubiquitin ligase with a neo-substrate protein to ubiquitinate and degrade the target through the proteasome.^1,2^ Molecular glue degraders are potentially more promising compared to heterobifunctional Proteolysis Targeting Chimeras (PROTACs) because of their lower molecular weights and associated drug-like properties, as well as their potential to exploit shallow protein–protein interfaces between an E3 ligase and less tractable therapeutic proteins that may not possess deep binding pockets.^1^ However, most molecular glue degraders have either been discovered fortuitously or through phenotypic screens.^1,3−7^ Rational chemical design of molecular glue or monovalent degraders in a target-based manner remains challenging.

Many recent studies have reported how subtle chemical alterations to otherwise nondegradative small-molecule inhibitors converted them into molecular glue degraders of their respective targets.^6−9^ These studies gave rise to the exciting possibility of transplantable chemical handles that could be appended onto the exit vector of protein-targeting ligands to convert these compounds into molecular glue degraders of their targets. E3 ligases have been shown to be ligandable with covalent small-molecules and chemoproteomic approaches.^10−16^ Covalent handles have also been successfully used in heterobifunctional PROTACs to identify permissive chemical handle and ligandable E3 ligase pairs that can be exploited for targeted protein degradation applications. These studies have identified various covalent handles targeting cysteines in E3 ligase substrate receptors DCAF16 and DCAF11.^13,17,18^ Covalent ligand screens against specific ubiquitin proteasome system components have also yielded new E3 ligase, E2 ubiquitin conjugating enzyme, or Cullin adaptor recruiters against RNF114, RNF4, FEM1B, UBE2D, DDB1, and SKP1 that can be used for PROTACs.^12,19−24^

Recent studies have also revealed that covalent chemistry can be used to identify potential chemical handles that enable the rational design of monovalent or molecular glue degraders. We previously discovered a covalent chemical handle that targets a cysteine in the quality control E3 ligase RNF126 that could be appended to the exit vector of a diverse range of protein-targeting ligands without the necessity for a linker to enable degradation of their respective targets.^25^ Covalent molecular glue degraders have also been discovered that enhance weak existing interactions between DCAF16 and BRD4 to degrade BRD4 in a template-assisted covalent modification approach.^26,27^

In this study, we sought to identify additional transplantable covalent chemical handles that can convert nondegradative inhibitors into molecular glue or monovalent degraders of their respective targets. We have identified a vinylsulfonyl piperazine handle that acts through targeting a cysteine within DCAF16 to not only enable the degradation of BRD4, but also several additional neo-substrates.

## Results

### Identifying Covalent Handles That Enable the Degradation of BRD4

**Figure 1:**
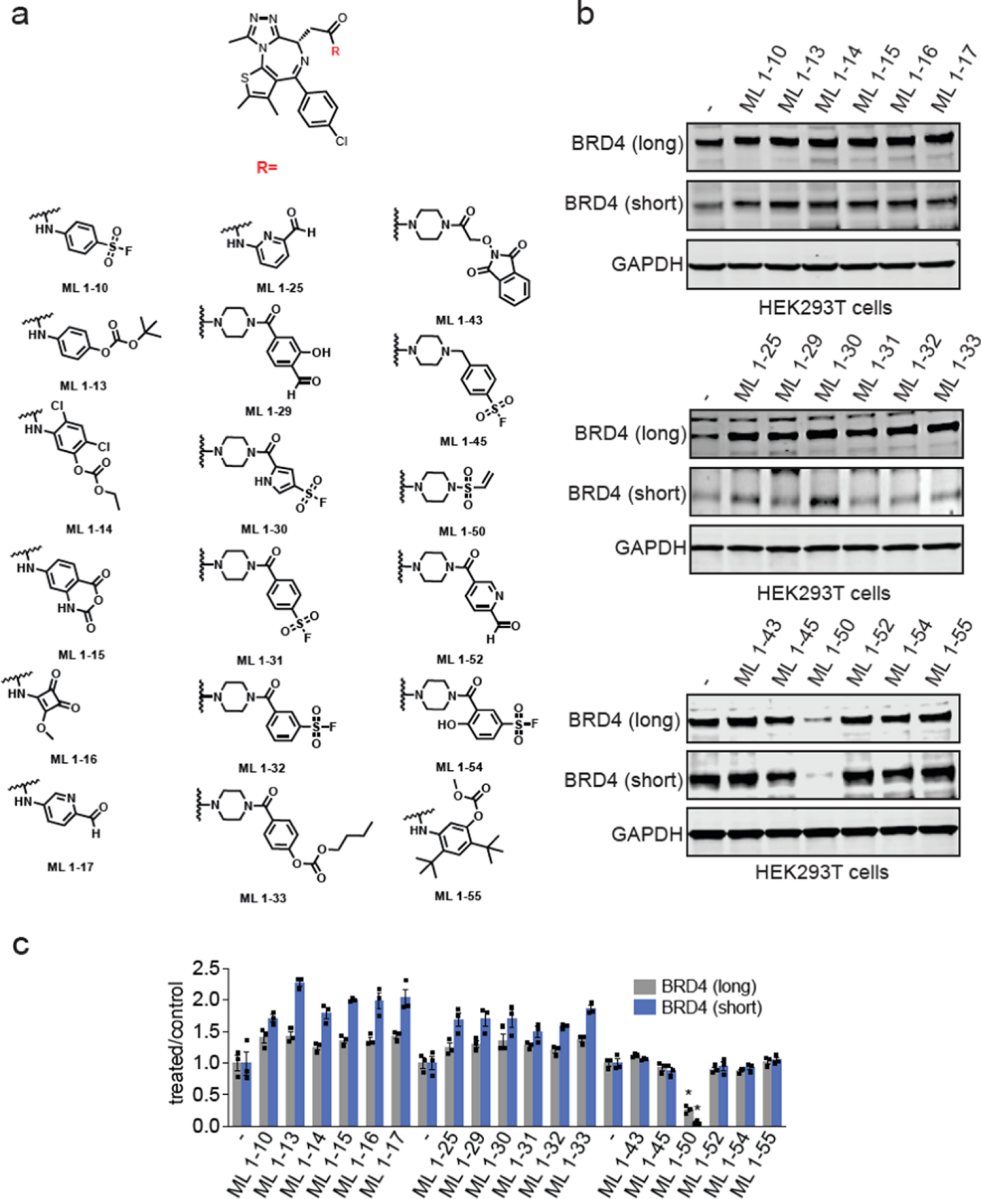
Identifying covalent handles that enable the degradation of BRD4. (a) Series of analogs of the BET family inhibitor JQ1 bearing various electrophilic handles. (b) Testing for BRD4 degradation with covalent JQ1 derivatives. HEK293T cells were treated with DMSO vehicle or covalent JQ1 derivatives (10 μM) for 24 h and BRD4 long and short isoforms and GAPDH loading control levels were assessed by Western blotting. Shown are gels that are representative of *n* = 3 biologically independent replicates per group. (c) Quantitation of BRD4 long and short isoforms from experiment described in (b) showing individual replicate values and average ± sem. Significance is expressed as *p < 0.001 compared to vehicle-treated controls.

**Figure 2:**
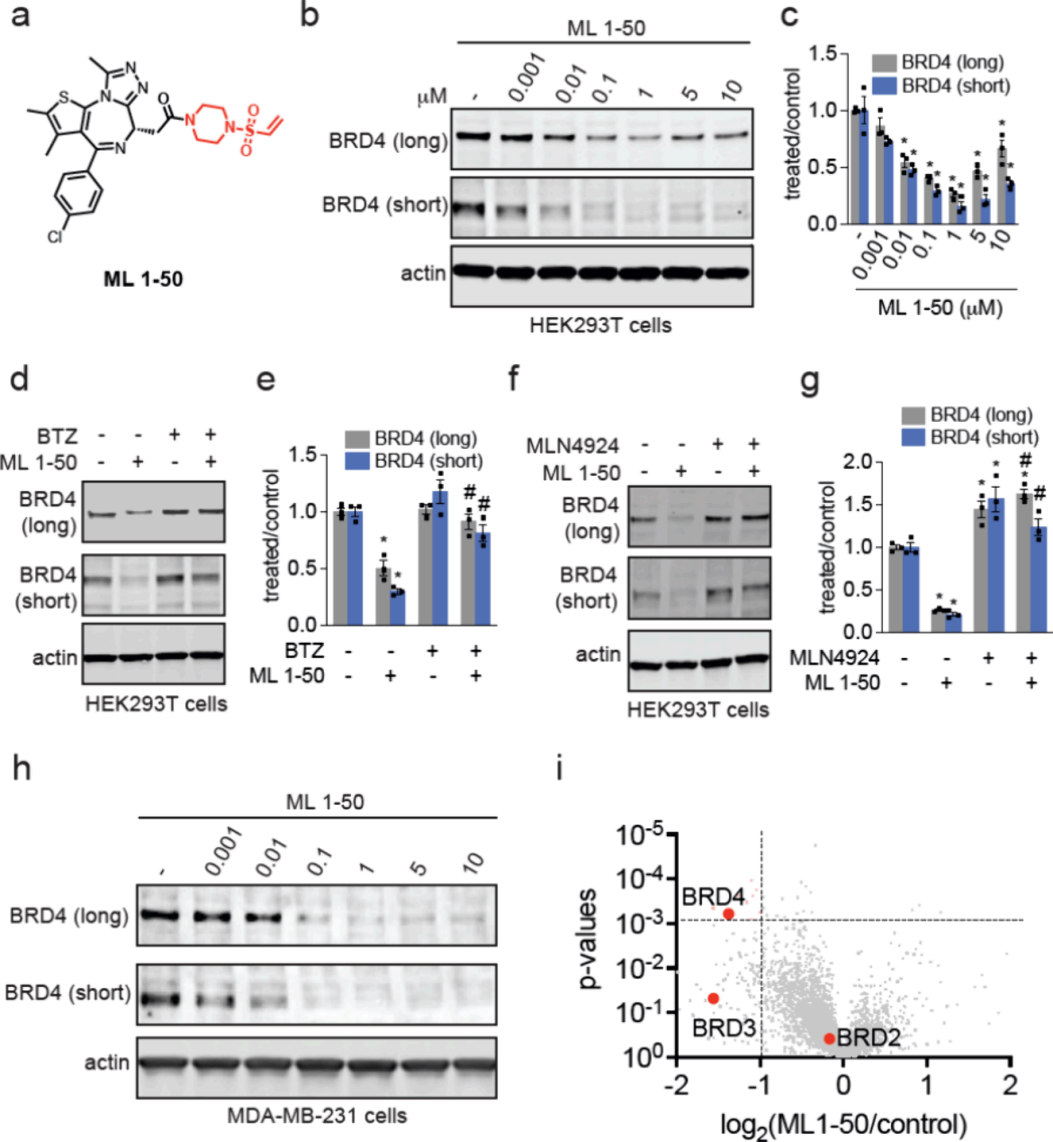
Characterization of the monovalent and covalent BRD4 degrader ML 1–50. (a) Structure of ML 1–50 with the vinylsulfonyl piperazine covalent chemical handle in red. (b, c) Dose–response of BRD4 degradation. HEK293T cells were treated with DMSO vehicle or ML 1–50 for 24 h. BRD4 and actin loading control levels were assessed by Western blotting and quantified in (c). (d, e) Proteasome inhibitor attenuation of BRD4 degradation. HEK293T cells were pretreated with DMSO vehicle or bortezomib (1 μM) 1 h prior to DMSO vehicle or ML 1–50 (1 μM) treatment for 24 h. BRD4 and actin loading control levels were assessed by Western blotting and quantified in (e). (f, g) NEDD8 activating enzyme inhibitor attenuation of BRD4 degradation. HEK293T cells were pretreated with DMSO vehicle or MLN4924 (1 μM) 1 h prior to DMSO vehicle or ML 1–50 (1 μM) treatment for 24 h. BRD4 and actin loading control levels were assessed by Western blotting and quantified in (g). (h) BRD4 degradation in MDA-MB-231 cells. MDA-MB-231 cells were treated with DMSO vehicle or ML 1–50 for 24 h and BRD4 and actin loading control levels were assessed by Western blotting. (i) Tandem mass tagging (TMT)-based quantitative proteomic profiling of ML 1–50 in MDA-MB-231 cells. MDA-MB-231 cells were treated with DMSO vehicle or ML 1–50 (1 μM) for 24 h. Proteins that were lowered in levels by >2-fold with *p* < 0.001 are highlighted in red with BRD4 specifically labeled, alongside other JQ1 targets BRD2 and BRD3. Data are from *n* = 3 biologically independent replicates per group. Blots shown in (b, d, f, h) are representative of *n* = 3 biologically independent replicates per group. Bar graphs in (c, e, g) show average ± sem. Significance is expressed as *p < 0.05 compared to vehicle-treated controls and #*p* < 0.05 compared to ML 1–50 treatment alone.

To identify covalent chemical handles that could convert nondegradative inhibitors into molecular glue or monovalent degraders of their targets, we used the BET family inhibitor JQ1 as a testbed to generate a series of covalent JQ1 analogs bearing various electrophilic handles that could react with cysteines, lysines, or other nucleophilic amino acids on E3 ligases to degrade BRD4 (Figure 1a). We synthesized and tested 18 derivatives. Among these compounds, only one compound, ML 1–50, led to the loss of both the long and short isoforms of BRD4 in HEK293T cells (Figure 1b-1c; Figure 2a). ML 1–50 reduced BRD4 levels in a dose-dependent manner with preferential degradation of the short BRD4 isoform with nanomolar potency in HEK293T cells (Figure 2b-2c). We observed hook effects with degradation of the long BRD4 isoform. ML 1–50 only showed modest cell viability impairments in HEK293T cells at the highest concentration of 10 μM tested (Figure S1a). This BRD4 degradation was attenuated by pretreatment with either proteasome inhibitor or NEDD8-activating enzyme inhibitor MLN4924 demonstrating that the loss of BRD4 was dependent on the proteasome and also a Cullin E3 ubiqutin ligase, respectively (Figure 2d-2g). We had previously observed preference for degradation of the long versus short isoforms of BRD4 with covalent PROTACs that appeared be specific to HEK293T cells compared to other cell lines.^23,24^ Similarly, we found that ML 1–50 potently degraded both the long and short BRD4 isoforms in the MDA-MB-231 breast cancer cell line, with no hook effects observed (Figure 2h, Figure S1b). Quantitative proteomic profiling of ML 1–50 in MDA-MB-231 cells showed relatively selective BRD4 degradation with only 11 other proteins that were significantly reduced in levels by greater than 2-fold (Figure 2i; Table S1). These 11 targets that are degraded are likely due to off-targets of our covalent degradative handle, which still requires further medicinal chemistry efforts to improve selectivity. These 11 targets included KIAA0101, RSBN1L, CCCNB1, MND1, UBE2C, RTFDC1, FAM204A, KIFC1, and IRS1 (Table S1). JQ1 also engages BRD2 and BRD3 and JQ1-based PROTACs have led to the degradation of BRD2, BRD3, and BRD4.^28^ With ML 1–50, we did not observe BRD2 degradation, but we did observe loss of BRD3 below our statistical significance threshold (Figure 2i; Table S1).

### Mapping the E3 Ligase Responsible for BRD4 Degradation

**Figure 3:**
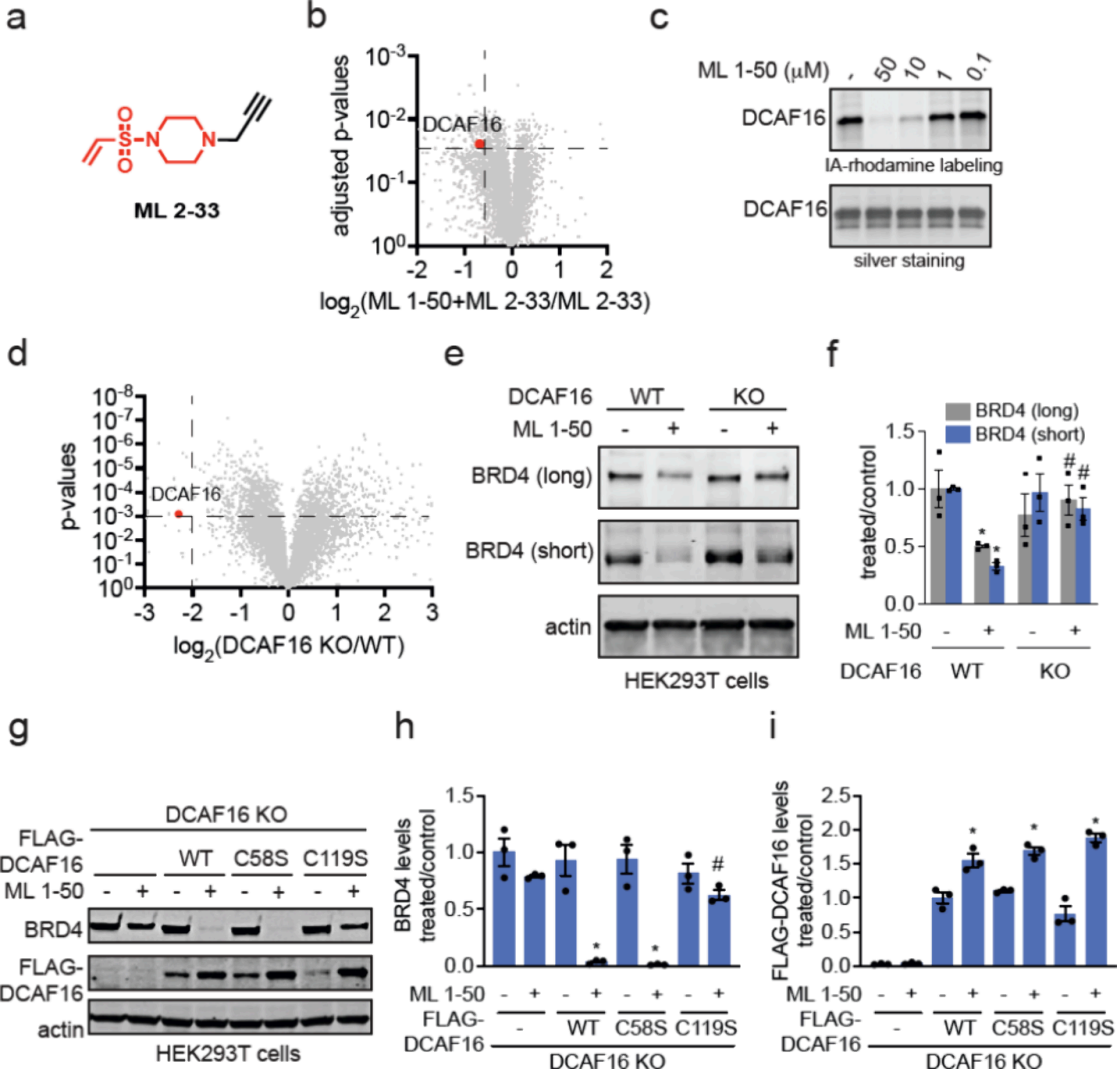
Identifying the E3 ligase responsible for ML 1–50-mediated BRD4 degradation. (a) Structure of alkyne-functionalized probe of the vinylsulfonyl piperazine handle (highlighted in red). (b) ML 1–50-outcompeted targets enriched by ML 2–33. HEK293T cell lysate were pretreated with DMSO vehicle or ML 1–50 (200 μM) 1 h prior to treatment with the ML 2–33 probe (20 μM). Probe-modified proteins were subjected to copper-catalyzed azide alkyne cycloaddition (CuAAC) with an azide-functionalized biotin enrichment handle. Probe-modified proteins were avidin-enriched, tryptically digested, and analyzed by TMT-based proteomics. Among the significantly outcompeted targets, DCAF16 highlighted in red was the only Cullin E3 ligase substrate receptor identified. (c) Gel-based ABPP of ML 1–50 against pure DCAF16. Pure DCAF16 protein was preincubated with DMSO vehicle or ML 1–50 for 30 min prior to addition of a rhodamine-functionalized cysteine-reactive iodoacetamide probe (IA-rhodamine) (250 nM) for 1 h. Proteins were resolved by SDS/PAGE and assessed by in-gel fluorescence and protein loading was assessed by silver staining. (d) TMT-based quantitative proteomic analysis of DCAF16 wild-type (WT) versus knockout (KO) cells. Because there was no commercial DCAF16 antibody, proteomic methods were used to confirm DCAF16 knockout. DCAF16 is labeled in red. (e, f) BRD4 degradation in DCAF16 WT and KO HEK293 cells. DCAF16 WT and KO cells were treated with DMSO vehicle or ML 1–50 (1 μM) for 24 h and BRD4 and actin loading control levels were assessed by Western blotting and quantified in (f). (g) ML 1–50-mediated BRD4 degradation in DCAF16 KO HEK293 cells expressing WT, C58S, or C119S mutant FLAG-WT, C58S, or C119S DCAF16 was lentivirally and stably expressed in DCAF16 KO cells after which cells were treated with DMSO vehicle or ML 1–50 (1 μM) for 24 h and BRD4, FLAG-DCAF16, and loading control actin levels were assessed by Western blotting. (h, i) BRD4 and FLAG-DCAF16 levels from (g) were quantified. Proteomics experiments and blots in (b–i) are from *n* = 3 biologically independent replicates per group and blots are representative. Bar graphs in (f, h, i) show individual replicate values average ± sem. Significance is expressed as *p < 0.05 compared to vehicle-treated controls and #*p* < 0.05 compared to ML 1–50 treated DCAF16 WT cells in (f) and compared to ML 1–50 treated FLAG-DCAF16 WT-expressing cells in (h, i).

We next sought to identify the E3 ligase responsible for the BRD4 degradation observed with ML 1–50. We synthesized an alkyne-functionalized probe based on the vinylsulfonyl piperazine handle, ML 2–33 (Figure 3a). Given the simplicity of this handle without a more elaborated ligand attached, we surmised that the handle would likely be more promiscuous compared to ML 1–50. We thus performed a competitive pulldown chemoproteomic experiment from ML 2–33-treated cells searching for proteins that were significantly outcompeted by ML 1–50 pretreatment. The ML 2–33 pulldown proteomics experiment yielded 6259 proteins. Out of these proteins, we observed 133 proteins that were significantly out-competed by excess ML 1–50 (200 μM) (*p* < 0.03, with log_2_ less than −0.6) spanning diverse protein classes (Figure 3b; Table S2). Among these outcompeted targets, there was only one E3 ligase that was part of the Cullin E3 ligase family that could be regulated by NEDD8—DCAF16 (Figure 3b; Table S2). Using competitive activity-based protein profiling (ABPP), competing *in situ* ML 1–50 target binding against *ex situ* proteome-wide cysteine labeling with a cysteine-reactive probe, we showed significant engagement of C119 of DCAF16 in cells with a control versus ML 1–50 treated ratio of the probe-modified tryptic peptide of 1.4, indicating a ∼ 28% engagement of this cysteine (Figure S1c; Table S3). This modest degree of E3 ligase engagement is consistent with previous reports with covalent PROTACs showing that only a small fraction of the E3 ligase needs to be engaged to enable degradation of target proteins.^12,13,17^ Site of modification analysis by liquid chromatography–mass spectrometry (LC-MS/MS) on tryptic digests of pure DCAF16 labeled with ML 1–50 also showed a single labeled site on C119 (Figure S1d). Our ABPP analysis identified 32 cysteines that were significantly engaged by >80% among >10,000 cysteines profiled (Table S3). Interestingly, there was no overlap between the targets identified in ABPP versus ML 2–33 competitive pulldown experiments. While our chemoproteomic data collectively demonstrated that ML 1–50 likely possesses a significant number of off-targets and still requires further medicinal chemistry optimization, we sought to further characterize the role of DCAF16 in ML 1–50 mediated BRD4 degradation effects.

To further confirm direct interaction of ML 1–50 with DCAF16, we showed that ML 1–50 displaced cysteine-reactive probe labeling of pure DCAF16 protein by gel-based ABPP approaches without causing any precipitation of the protein (Figure 3c). We further demonstrated that the ML 1–50-mediated degradation of BRD4 could be outcompeted with pretreatment of cells with excess JQ1 and that treatment of cells with the covalent alkyne-functionalized handle ML 2–33 alone did not alter BRD4 levels, indicating that the loss of BRD4 was likely through specific interactions with BRD4 and not through nonspecific effects of the covalent handle (Figure S1e–S1g). Consistent with the role of DCAF16 in our observed effects, we showed that the BRD4 degradation from ML 1–50 treatment was significantly and completely attenuated in DCAF16 knockout cells compared to wild-type cells (Figure 3d-3f; Table S4). Previous reports have identified a covalent molecular glue degrader between DCAF16 and BRD4, MMH2, in which the covalent warhead was attached to an orthogonal exit vector from JQ1, compared to our ML 1–50 compound.^29^ Interestingly, MMH2 targeted C58 of DCAF16.^29^ In this structure, C119 did not appear to be solvent exposed. Zhang and Cravatt et al. also demonstrated that their covalent DCAF16-based PROTACs acted through yet another set of cysteines, C177 and/or C179.^13^ As such, we were skeptical whether our covalent degraders really acted through targeting yet another cysteine on DCAF16—C119. As such, we expressed FLAG-DCAF16 WT, C58S, or C119S in DCAF16 knockout cells to determine which mutant may confer resistance to ML 1–50-mediated BRD4 degradation. Consistent with our site-of-modification analysis, we demonstrated that expression of the C119S DCAF16 mutant, but not the C58S mutant, conferred significant resistance to ML 1–50-mediated BRD4 degradation observed in DCAF16 WT-expressing cells (Figure 3g-3i). Interestingly, we also observed that ML 1–50 treatment consistently increased FLAG-DCAF16 protein levels even in the C119S mutant line, potentially suggesting that the previously reported weak ligand-induced interactions between DCAF16 and BRD4^9,29^ may help to stabilize DCAF16 protein expression (Figure 3g,3i).

**Figure 4:**
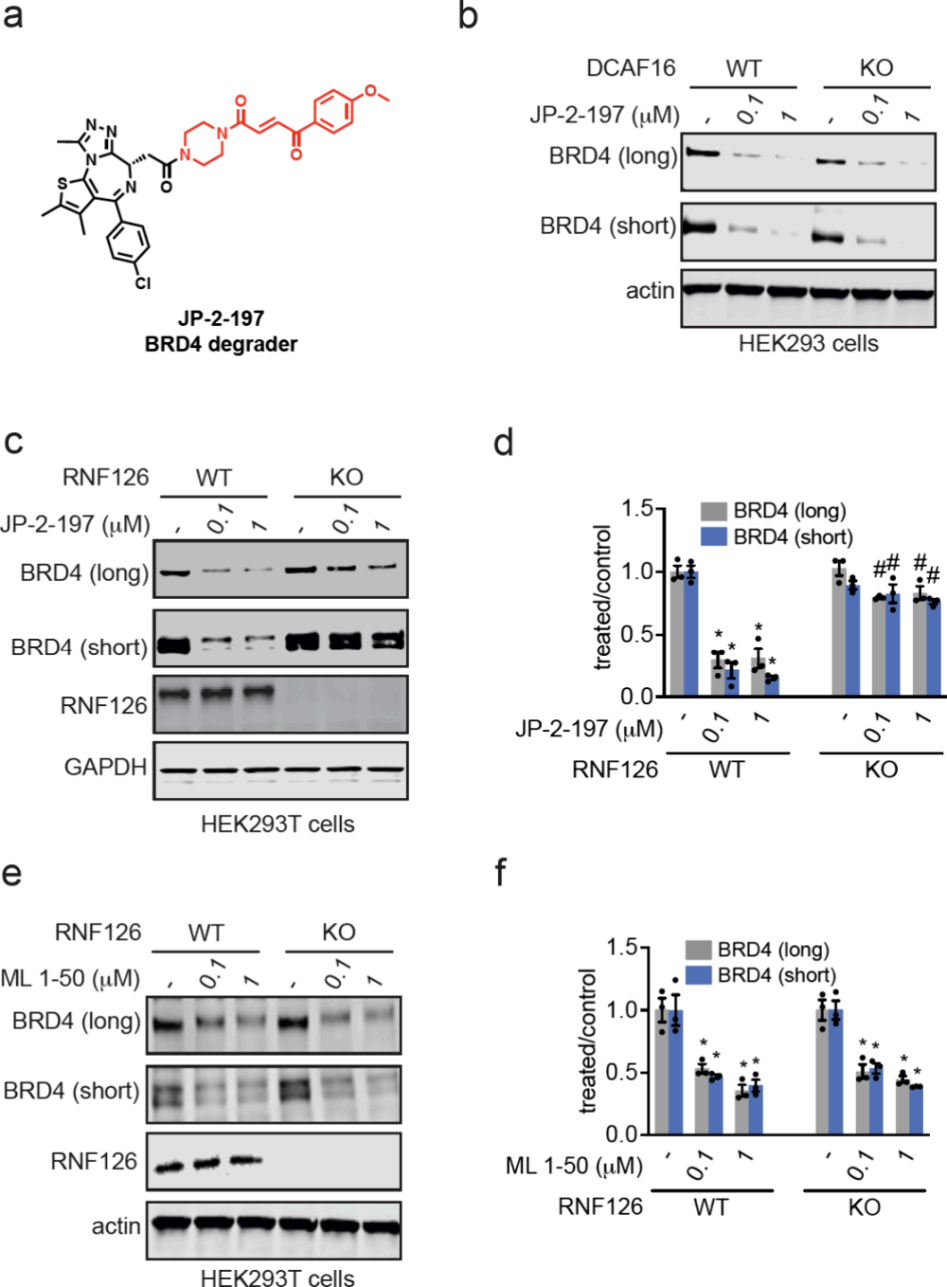
Testing the dependence of covalent monovalent BRD4 degraders on DCAF16 versus RNF126. (a) Structure of our previously published covalent monovalent BRD4 degrader JP-2–197 bearing the covalent “fumarate” handle shown in red. (b) BRD4 degradation in DCAF16 WT and KO HEK293 cells. DCAF16 WT and KO HEK293 cells were treated with DMSO vehicle or JP-2–197 for 24 h and BRD4 and actin loading control levels were assessed by Western blotting. (c, d) BRD4 degradation in RNF126 WT and KO cells. RNF126 WT and KO HEK293T cells were treated with JP-2–197 for 10 h and BRD4, RNF126, and GAPDH loading control levels were assessed by Western blotting and quantified in (d). (e, f) BRD4 degradation in RNF126 KO HEK293T cells. RNF126 WT and KO HEK293T cells were treated with ML 1–50 for 16 h and BRD4, RNF126, and actin loading control levels were assessed by Western blotting and quantified in (f). Blots in (b, c, e) are representative of *n* = 3 biologically independent replicates per group. Bar graph in (d, f) shows individual replicate values and average ± sem. Significance is expressed as *p < 0.05 compared to vehicle-treated controls and #*p* < 0.05 compared to JP-2–197 or ML 1–50 treated RNF126 WT cells.

Given that several previous studies have identified DCAF16 as the E3 ligase substrate receptor responsible for the degradation of covalent BRD4 degraders bearing various different types of electrophilic handles, in part because of the native weak affinity between BRD4 and DCAF16,^13,18,26,27^ we next determined whether our previously discovered covalent monovalent BRD4 degrader, JP-2–197, bearing a but-2-ene, 1,4-dione “fumarate” covalent degrader handle that targets RNF126 instead acts through DCAF16.^25^ We showed that the BRD4 degradation observed by JP-2–197 was not mitigated at all in DCAF16 knockout cells (Figure 4a-4b). In contrast, we observed complete and significant attenuation of BRD4 degradation in RNF126 knockout cells (Figure 4c-4d). Furthermore, we demonstrated that ML 1–50-mediated degradation of BRD4 is not diminished in RNF126 KO cells compared to WT cells (Figure 4e-4f). Our data thus indicate that different electrophilic degraders against the same target can act through distinct E3 ligases.

### Exploring the Applicability of the Covalent Handle against Other Target Proteins

**Figure 5:**
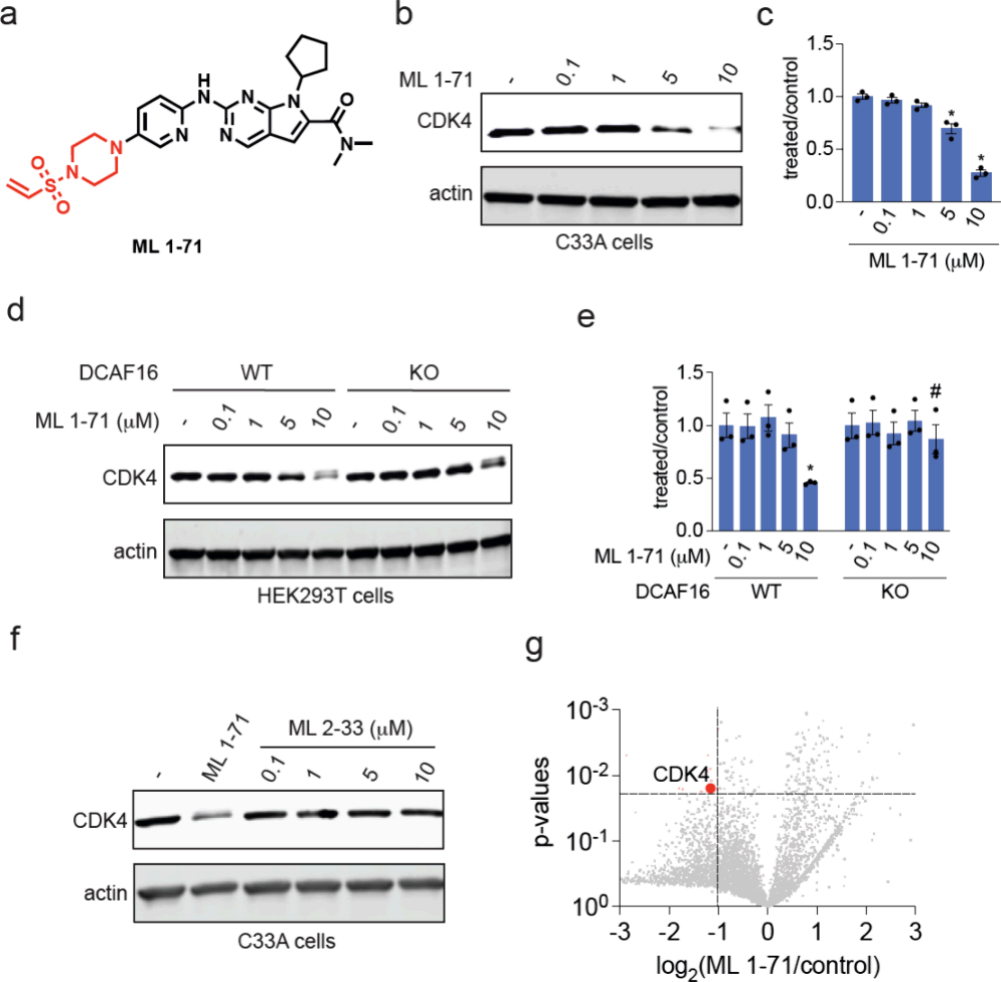
Characterization of CDK4 monovalent degrader. (a) Structure of ML 1–71, a CDK4 inhibitor ribociclib bearing a vinylsulfonyl piperazine handle highlighted in red. (b, c) CDK4 degradation in C33A cervical cancer cells. C33A cells were treated with DMSO vehicle or ML 1–71 for 24 h and CDK4 and actin loading control levels were assessed by Western blotting and quantified in (c). (d, e) CDK4 degradation in DCAF16 WT and KO HEK293 cells. DCAF16 WT and KO HEK293 cells were treated with DMSO vehicle or ML 1–71 for 24 h and CDK4 and actin loading control levels were assessed by Western blotting and quantified in (e). (f) CDK4 levels in C33A cells. C33A cells were treated with DMSO vehicle, ML 1–71 (10 μM), or ML 2–33 for 24 h and CDK4 and loading control actin levels were assessed by Western blotting. (g) TMT-based quantitative proteomic profiling of ML 1–71 in C33A cells. C33A cells were treated with DMSO vehicle or ML 1–71 (10 μM) for 24 h. Proteins that were reduced in levels by >2-fold with *p* < 0.05 are designated in red with CDK4 labeled. Data are from *n* = 3 biologically independent replicates per group. Blots in (b, d, f) are representative of *n* = 3 biologically independent replicates per group. Bar graph in (e) shows individual replicate values and average ± sem. Significance is expressed as *p < 0.05 compared to vehicle-treated controls and ^#^*p* < 0.05 compared to ML 1–71 treated DCAF16 WT cells.

While we identified a covalent handle that can convert the nondegradative JQ1 into a degrader of BRD4, BRD4 is extremely susceptible to targeted protein degradation and thus demonstrating proof-of-concept of a covalent handle that can degrade BRD4 does not speak to the broader applicability of this handle for other targets. Furthermore, previous studies have already demonstrated covalent and noncovalent BRD4 molecular glue degraders that act through DCAF16, through strengthening already existing weak interactions between DCAF16 and BRD4.^8,9,26,29^ Previous studies have also used covalent DCAF16 recruiters to generate heterobifunctional PROTACs against other targets beyond BRD4.^13^ Whether a DCAF16-targeting covalent handle could be broadly transplanted across other protein-targeting ligands to generate linker-less monovalent degraders of neo-substrate proteins beyond BRD4 is unknown. We first appended the vinylsulfonyl piperazine handle onto the clinically approved CDK4/6 inhibitor ribociclib to generate ML 1–71 (Figure 5a). ML 1–71 significantly degraded CDK4 in cells in a dose-dependent manner, albeit less potently compared to ML 1–50 and BRD4 (Figure 5b-5c). Despite the modest potency of this degrader, we still observed significant attenuation of CDK4 degradation in DCAF16 knockout cells (Figure 5d-5e). We also did not observe degradation of CDK4 with ML 2–33 treatment and did not observe cytotoxicity with ML 1–71 treatment (Figure 5f; Figure S2). Quantitative proteomic profiling of ML 1–71 in C33A cervical cancer cells also demonstrated relatively selective CDK4 degradation with only 15 other targets reduced in levels that may arise from transcriptional effects downstream of CDK4 inhibition or off-target effects (Figure 5g; Table S5). These targets included CHEK1, PSMG1, STK4, KEAP1, FASN, GART, FGD6, AASDHPPT, PBK, MRTO4, TUBB2B, DUS3L, TUBB4A, MAP2K4. Given that CHEK1, STK4, PBK, and MAP2K4 are kinases, perhaps these may be off-targets of the CDK4/6 inhibitor ribociclib. We next generated a monovalent degrader for SMARCA2/4 bearing the vinylsulfonyl piperazine handle—ML 1–96 (Figure S3a).^30^ ML 1–96 significantly degraded both SMARCA2 and SMARCA4 in MV-4–11 leukemia cancer cells (Figure S3b-S3c). ML 1–96-mediated SMARCA2/4 degradation was attenuated in DCAF16 knockout cells (Figure S3d-S3e). We also demonstrated that ML 2–33 treatment does not affect SMARCA2/4 levels and ML 1–96 was not cytotoxic to cells (Figure S3f-S3g).

**Figure 6:**
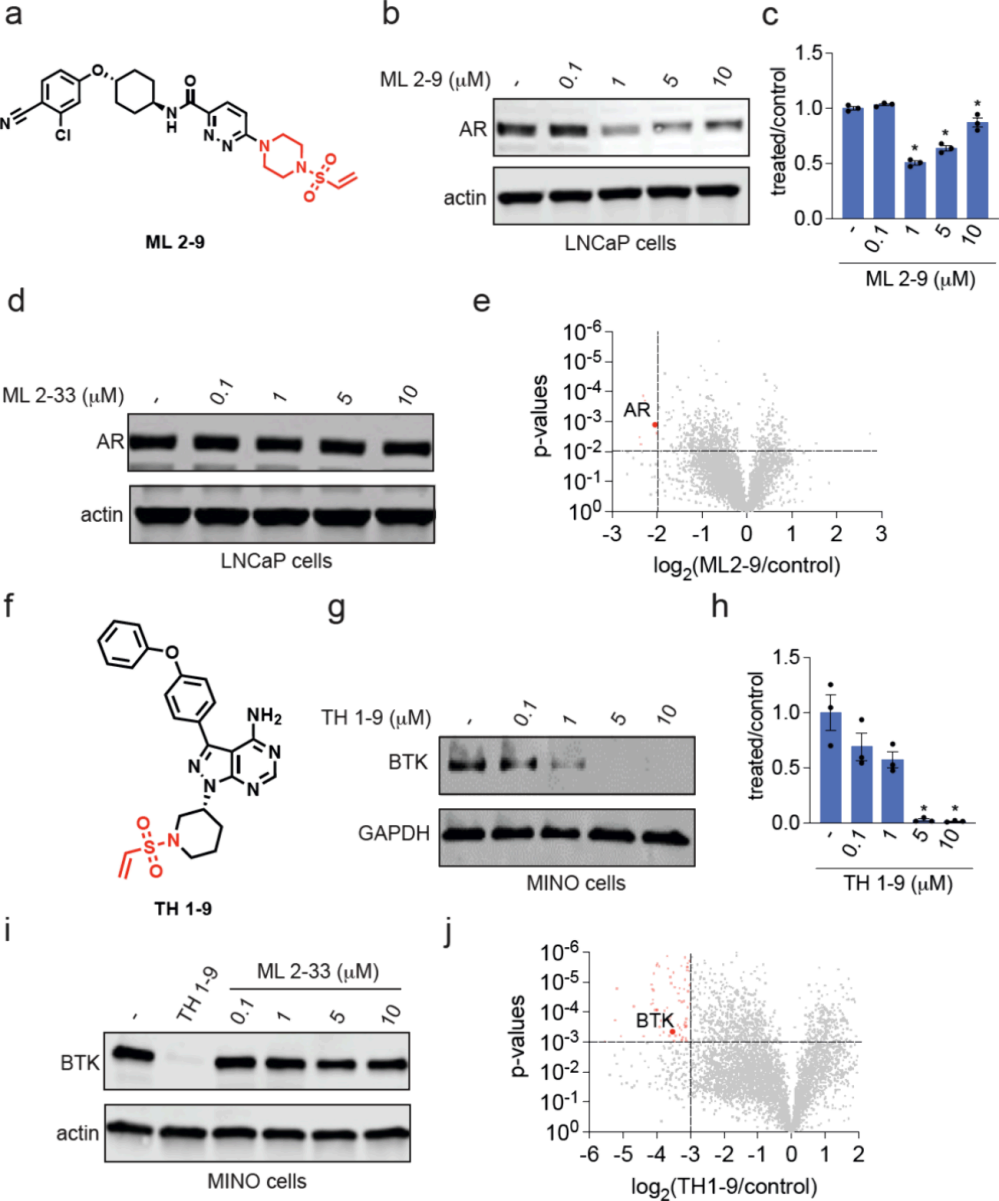
Characterization of AR and BTK monovalent degraders. (a) Structure of AR monovalent degrader ML 2–9 with AR-targeting ligand derived from the ARV-110 PROTAC bearing the covalent vinylsulfonyl piperazine handle highlighted in red. (b, c) AR degradation in LNCaP prostate cancer cells. LNCaP cells were treated with DMSO vehicle or ML 2–9 for 24 h and AR and actin loading control levels were assessed by Western blotting and quantified in (c). (d) AR levels in LNCaP cells. LNCaP cells were treated with DMSO vehicle or ML 2–33 for 24 h and AR and loading control actin levels were assessed by Western blotting. (e) TMT-based quantitative proteomic profiling of ML 2–9 in LNCaP cells. LNCaP cells were treated with DMSO vehicle or ML 2–9 (1 μM) for 24 h. Proteins that were reduced in levels by >4-fold with *p* < 0.01 are designated in red with AR labeled. Data are from *n* = 3 biologically independent replicates per group. (f) Structure of BTK monovalent degrader TH 1–9 with BTK inhibitor derived from ibrutinib bearing the covalent vinylsulfonyl piperazine handle highlighted in red. (g, h) BTK degradation in MINO lymphoma cancer cells. MINO cells were treated with DMSO vehicle or TH 1–9 for 24 h and BTK and GAPDH loading control levels were assessed by Western blotting and quantified in (h). (i) BTK levels in MINO cells. MINO cells were treated with DMSO vehicle, TH1–9 (10 μM), or ML 2–33 for 24 h and BTK and loading control actin levels were assessed by Western blotting. (j) TMT-based quantitative proteomic profiling of TH 1–9 in MINO cells. MINO cells were treated with DMSO vehicle or TH 1–9 (5 μM) for 24 h. Proteins that were reduced in levels by >8-fold with *p* < 0.001 are designated in red with BTK labeled. Data are from *n* = 3 biologically independent replicates per group. Blots in (b, d, g, f) are representative of *n* = 3 biologically independent replicates per group. Bar graphs in (c, h) show individual replicate values and average ± sem. Significance is expressed as *p < 0.05 compared to vehicle-treated controls.

To further explored the substrate scope of our covalent degradative handle, we next generated an androgen receptor (AR) monovalent degrader consisting of the AR-targeting ligand from the AR PROTAC ARV-110 and the vinylsulfonyl piperazine handle—ML 2–9 (Figure 6a).^31^ ML 2–9 significantly degraded AR in LNCaP prostate cancer cells (Figure 6b-6c). ML 2–33 did not alter AR levels (Figure 6d). Interestingly, a hook effect was observed with this degrader. Proteomic data showed selective AR degradation with only 7 other proteins reduced in levels (Figure 6e; Table S6). These proteins included ADCY5, CDK1, MID1PIP1, KLHDC2, SBNO1, and FBXO28 (Table S6). We also demonstrated that ML 2–9 did not show any cytotoxicity in LNCaP prostate cancer cells (Figure S4a). Unfortunately, we could not determine DCAF16 dependence because DCAF16 knockdown impaired cell viability of LNCaP cells, indicating that DCAF16 may be essential in this particular cell line. We also made a vinylsulfonyl piperazine bearing derivative of the BCR-ABL and c-ABL kinase inhibitor dasatinib, ML 2–5, and demonstrated the degradation of both the fusion oncogene and the parent kinase in K562 leukemia cancer cells (Figure S4b-S4d). We once again demonstrated that ML 2–33 treatment did not alter BCR-ABL or c-ABL levels and ML 2–5 only caused modest cytotoxicity in K562 cells (Figure S4e-S4f). Importantly, DCAF16 knockdown significantly attenuated ML 2–5-mediated degradation of both BCR-ABL and c-ABL, demonstrating that this degrader was still dependent on DCAF16 (Figure S4g-S4i). Finally, we also generated a vinylsulfonyl derivative of the BTK inhibitor ibrutinib, TH 1–9, and showed that this compound also degrades BTK in dose-dependent manner in MINO lymphoma cancer cells (Figure 6f-6h). We demonstrated that ML 2–33 treatment did not affect BTK levels (Figure 6i). Proteomic analyses revealed a higher number of proteins, 73 off-targets in total, beyond BTK that were lowered in levels compared to the other degraders (Figure 6j; Table S7). This may be due to the many off-targets of ibrutinib^32^ at the high concentrations used in this study or may be due to downstream transcriptional changes resulting from BTK inhibition and degradation. The full list of the off-target proteins can be found in Table S7, but examples of off-targets included NOSIP, ALDH6A1, TOE1, SRSF9, SYK, CLK3, TLK2, DUS3L, KEAP1, CDK4, CDKN2A, and ADRBK1. Note that SYK, CLK3, TLK2, CDK4, and ADRBK1 are kinases and may potentially be off-targets of ibrutinib. Despite this moderate selectivity of degradation, we still observed significant partial attenuation of TH 1–9-mediated BTK degradation upon DCAF16 knockdown (Figure S5a-S5c). The incomplete rescue is likely due to partial DCAF16 knockdown in MINO cells. Overall, this degradative covalent handle thus enabled the degradation of not only BRD4, but also several other proteins, including CDK4, SMARCA2/4, AR, BTK, and BCR-ABL/c-ABL.

## Discussion

Overall, we have identified a covalent DCAF16 degradative handle that can be transplanted across a diverse range of protein targeting ligands, without the need for a linker, to enable the degradation of several different protein classes. We note that this is a proof-of-concept study further demonstrating the possibility of covalent chemical handle and permissive E3 ligase pairs that can potentially be exploited toward rationally designing monovalent or molecular glue degraders, beyond our previously discovered covalent RNF126-targeting degradative handle.^25^ While BRD4 has been shown to possess weak interactions with DCAF16, based on the UbiBrowser database, CDK4, SMARCA2/4, AR, BTK, and BCR-ABL/c-ABL do not appear to natively interact with DCAF16. As such, DCAF16 may have utility for both PROTACs^13^ and molecular glue degraders of neo-substrates beyond BRD4.

We note that the potency, selectivity, and pharmacokinetic properties of our covalent handle would need to be substantially improved for future translational efforts. Each of our degraders in this study still shows off-target degradation, likely indicative of either off-targets of the protein-targeting ligands at the concentrations used or off-target effects resulting from our covalent degradative handle. For example, we saw that KEAP1 and DUS3L were common off-targets degraded by the CDK4 and BTK degraders (Table S5, Table S7). While our genetic rescue studies demonstrate that the degradative on-target action of our degraders is resulting from DCAF16, we did observed that KEAP1 was likely a direct target of our covalent degradative handle from ML 2–33 pulldown chemoproteomic experiments, and that it did not pass our filtering criteria for ML 1–50-competed targets (Table S2). KEAP1 has cysteines that are sensitive to oxidants and electrophiles and we have previously shown that engagement of KEAP1 with bardoxolone-based PROTACs lowers KEAP1 levels in cells.^33^ As such, future medicinal chemistry efforts would be required to further improve the selectivity of our covalent degradative handle.

Our study, coupled with several other prior studies, also demonstrates the unique susceptibility of DCAF16 to covalent targeting for monovalent or heterobifunctional targeted protein degradation applications.^13,26,29^ This study also points to the potential permissiveness of DCAF16 in enabling the degradation of many different neo-substrate proteins, compared to other E3 ligases such as KEAP1 that may be much more restrictive in its substrate scope.^34^ Intriguingly, our results indicate that our covalent DCAF16-dependent degraders act through targeting C119 on DCAF16. This is in contrast to previously reported DCAF16-dependent PROTACs and molecular glue degraders that have been shown to act through C58 or C177/C179.^13,29^ The permissiveness of DCAF16 for targeted protein degradation of neo-substrates may be in-part due to the several different cysteines that can be exploited on DCAF16. Understanding whether these cysteines on DCAF16 may generally act as an electrophile sensor for degradation of native DCAF16 substrates or whether these cysteines may be regulated by cellular redox balance will be of future interest. Furthermore, based on DCAF16 structures previously solved with previously discovered DCAF16-based BRD4 molecular glue degraders,^9,29^ C119 does not appear to be solvent-exposed. This C119 on DCAF16 may become exposed when the other neo-substrate binding pockets where C58 or C177/C179 reside are not occupied. Future structural biology studies will be useful in understanding the accessibility of C119. We also demonstrate that not every electrophilic monovalent degrader acts through DCAF16. We show that our previously discovered covalent BRD4 degrader bearing a “fumarate” handle acts through RNF126, and not through DCAF16. Our results are analogous to recent findings with covalent heterobifunctional BRD4 degraders bearing two different electrophilic handles that act through DCAF16 and DCAF11.^18^ Our study also suggests that pre-existing weak interactions between the E3 ligase and protein target may not be necessary to develop monovalent degraders. We also acknowledge that our covalent degraders show independent binding to both the target protein and DCAF16, analogous to one of the original molecular glues rapamycin that has independent binding to FKBP12 and the FRB domain of mTORC1.^2,35,36^ While we have chosen to call our degraders monovalent degraders or molecular glue degraders, one may also choose to classify these degraders as bivalent degraders or “mini-PROTACs” wherein the piperazine could be viewed as the linker with a minimum degradative covalent handle. Although not observed with most of our degraders, this may explain the “hook effect” observed with our AR degrader. Overall, our study underscores the utility of covalent chemoproteomic approaches in identifying covalent degradative handle and permissive E3 ligase pairs to expand the scope of targeted protein degradation applications.

## Methods

### Cell Culture

HEK293T and HEK293 cells were obtained from the UC Berkeley Cell Culture Facility and were cultured in Dulbecco’s Modified Eagle Medium (DMEM) containing 10% (v/v) fetal bovine serum (FBS) and maintained at 37 °C with 5% CO_2_. C33A cells were purchased from the American Type Culture Collection (ATCC) and were cultured in DMEM containing 10% (v/v) FBS and maintained at 37 °C with 5% CO_2_. K562 cells were obtained from the UC Berkeley Cell Culture Facility and were cultured in Iscove’s Modified Dulbecco’s Medium (IMDM) containing 10% (v/v) FBS and maintained at 37 °C with 5% CO_2_. MV-4–11 cells were obtained from the ATCC and were cultured in IMDM containing 10% (v/v) FBS and maintained at 37 °C with 5% CO_2_. Mino cells were obtained from the ATCC and were cultured in RPMI-1640 Medium containing 10% (v/v) FBS and maintained at 37 °C with 5% CO_2_. LNCaP cells were obtained from the UC Berkeley Cell Culture Facility and were cultured in DMEM containing 10% (v/v) FBS and maintained at 37 °C with 5% CO_2_. HEK293 DCAF16 knockout cells were purchased from Ubigene Biosciences and were cultured in DMEM containing 10% (v/v) FBS and maintained at 37 °C with 5% CO_2_. Unless otherwise specified, all cell culture materials were purchased from Gibco. It is not known whether HEK293T cells are from male or female origin.

### Western Blotting

Cells were washed twice with cold PBS, scraped, and pelleted by centrifugation (1,200 g, 5 min, 4 °C). Pellets were resuspended in PBS, lysed by sonication or RIPA lysis buffer (Thermo Scientific), clarified by centrifugation (12,000 g, 10 min, 4 °C), and lysate was transferred to new low-adhesion microcentrifuge tubes. Proteome concentrations were determined using the BCA assay and lysate was diluted to appropriate working concentrations. Proteins were resolved by SDS/PAGE and transferred to nitrocellulose membranes using the Trans-Blot Turbo transfer system (Bio-Rad). Membranes were blocked with 5% BSA in Tris-buffered saline containing Tween 20 (TBS-T) solution for 1 h at RT, washed in TBS-T, and probed with primary antibody diluted in recommended diluent per manufacturer overnight at 4 °C. After 3 washes with TBS-T, the membranes were incubated in the dark with IR680- or IR800-conjugated secondary antibodies at 1:10,000 dilution in 5% BSA in TBS-T at RT for 1 h. After 3 additional washes with TBST, blots were visualized using an Odyssey Li-Cor fluorescent scanner. The membranes were stripped using ReBlot Plus Strong Antibody Stripping Solution (EMD Millipore, 2504) when additional primary antibody incubations were performed. Antibodies used in this study were BRD4 (Abcam ab128874), CDK4 (Abcam ab108357), GAPDH (Cell Signaling Technology 14C10), Beta Actin (Cell Signaling Technology 13E5), c-Abl (Santa Cruz Biotechnology sc-23), SMARCA2 (Abcam ab240648), BRG1 (SMARCA4) (Cell Signaling Technology D1Q7F), BTK (Cell Signaling Technology D3H5), Androgen Receptor (Cell Signaling Technology D6F11).

### Bortezomib or MLN4924 Rescue Studies

2E6 of HEK293T cells per 3 mL of media were plated in 6 cm plates and left overnight to adhere. Cells were pretreated for 1 h with either Bortezomib (Cayman, C835F70) or MLN4924 (Tocris Bioscience, 649910) at a final concentration of 1 μM. Cells were then treated with ML1–50 until desired time point. Cells from both the supernatant and on the plate were harvested and assessed via Western blot.

### Cell Viability Assay

Cells were seeded in 96-well white plates overnight and then treated with DMSO vehicle control or degraders and incubated at 37 °C for 24 h. Cell viability assay was performed using CellTiter-Glo 2.0 reagent (Promega, G9241) according to manufacturer’s protocol. Luminescent signals were measured using the Tecan Spark Plate reader (30086376).

### Isotopic Desthiobiotin (isoDTB)-ABPP Cysteine Chemoproteomic Profiling of ML1–50

HEK293T cells were treated with either ML1–50 (10 μM) or DMSO for 2 h before cell collection and lysis. The proteome concentrations were determined using BCA assay and adjusted to 2 mg/mL. For each biological replicate, 2 aliquots of 1 mL of 2 mg/mL were used (i.e., 4 mg per condition). Each aliquot was treated with 20 μL of IA-alkyne (26.6 mg/mL in DMSO, 200 μM final concentration) for 1 h at RT. Two master mixes of the click reagents were prepared in the meanwhile, each containing 510 μL TBTA (0.9 mg/mL in 4:1 tBuOH/DMSO), 165 μL CuSO4 (12.5 mg/mL in H_2_O), 165 μL TCEP (14.0 mg/mL in H_2_O) and 160 μL of either heavy or light isoDTB tags (4 mg in DMSO, Click Chemistry Tools, 1565). The samples were then treated with 120 μL of the heavy (DMSO treated) or light (compound treated) master mix for 1 h at RT. After incubation, one light and one heavy labeled samples were combined and acetone-precipitated overnight at −20 °C. The samples were then centrifuged at 3,500 rpm for 10 min, acetone was removed, and the protein pellets resuspended in cold MeOH by sonication. The samples were centrifuged at 3,500 rpm for 10 min and MeOH was removed (repeated 3× in total). The pellets were dissolved in 600 μL urea (8 M in 0.1 M TEAB) by sonication and the urea concentration was then adjusted to 2 M by adding 1800 μL of TEAB (0.1 M). Two tubes containing solubilized proteins were combined, further diluted with 2400 μL 0.2% NP40 in PBS, and bound to high-capacity streptavidin agarose beads (200 μL/sample, ThermoFisher, 20357) for 1 h at RT with mixing. The beads were then centrifuged for 1 min at 1,000 g, the supernatant was removed, and the beads were washed 3 times with 0.1% NP40 in PBS, 3 times with PBS and 3 times with H_2_O. The samples were then resuspended in 8 M urea (600 μL in 0.1 M TEAB) and treated with DTT (30 μL, 31 mg/mL in H_2_O) for 45 min at 37 °C. They were then reacted with iodoacetamide (30 μL, 74 mg/mL in H_2_O) for 30 min at RT, followed by DTT (30 μL, 31 mg/mL in H_2_O) for 30 min at RT. The samples were diluted with 1800 μL TEAB (0.1 M), centrifuged for 1 min at 1,000 g, and the supernatant was removed. The beads were resuspended in 400 μL urea (2 M in 0.1 M TEAB), and trypsin (8 μL, 0.5 mg/mL) was added and incubated for 20 h at 37 °C. The samples were then diluted with 800 μL 0.1% NP40 in PBS and the beads were washed 3 times with 0.1% NP40 in PBS, 3 times with PBS, and 3 times with H_2_O. Peptides were then eluted with 0.1% formic acid in 50% acetonitrile (3 × 400 μL). The samples were then dried using a vacuum concentrator at 30 °C, resuspended in 300 μL 0.1% TFA in H_2_O, and fractionated using high pH reversed-phase peptide fractionation kits (ThermoFisher, 84868) according to the manufacturer’s protocol.

### IsoDTB-ABPP Mass Spectrometry Analysis

Mass spectrometry analysis was performed on an Orbitrap Eclipse Tribrid Mass Spectrometer with a High Field Asymmetric Waveform Ion Mobility (FAIMS Pro) Interface (Thermo Scientific) with an UltiMate 3000 Nano Flow Rapid Separation LCnano System (Thermo Scientific). Off-line fractionated samples (5 μL aliquot of 15 μL sample) were injected via an autosampler (Thermo Scientific) onto a 5 μL sample loop which was subsequently eluted onto an Acclaim PepMap 100 C18 HPLC column (75 μm x 50 cm, nanoViper). Peptides were separated at a flow rate of 0.3 μL/min using the following gradient: 2% buffer B (100% acetonitrile with 0.1% formic acid) in buffer A (95:5 water:acetonitrile, 0.1% formic acid) for 5 min, followed by a gradient from 2 to 40% buffer B from 5 to 159 min, 40 to 95% buffer B from 159 to 160 min, holding at 95% B from 160 to 179 min, 95% to 2% buffer B from 179 to 180 min, and then 2% buffer B from 180 to 200 min. Voltage applied to the nano-LC electrospray ionization source was 2.1 kV. Data was acquired through an MS1 master scan (Orbitrap analysis, resolution 120,000, 400–1800 *m*/*z*, RF lens 30%, heated capillary temperature 250 °C) with dynamic exclusion enabled (repeat count 1, duration 60 s). Data-dependent data acquisition comprised a full MS1 scan followed by sequential MS2 scans based on 2 s cycle times. FAIMS compensation voltages (CV) of −35, −45, and −55 were applied. MS2 analysis consisted of: quadrupole isolation window of 0.7 *m*/*z* of precursor ion followed by higher energy collision dissociation (HCD) energy of 38% with an orbitrap resolution of 50,000.

Data was extracted in the form of MS1 and MS2 files using Raw Converter (Scripps Research Institute) and searched against the Uniprot human database using ProLuCID search methodology in IP2 v.3-v.5 (Integrated Proteomics Applications, Inc.).^37^ Cysteine residues were searched with a static modification for carboxyaminomethylation (+57.02146) and up to two differential modifications for methionine oxidation and either the light or heavy isoDTB tags (+561.33872 or +567.34621, respectively). Peptides were required to be fully tryptic peptides. ProLuCID data were filtered through DTASelect to achieve a peptide false-positive rate below 5%. Only those probe-modified peptides that were evident across two out of three biological replicates were interpreted for their isotopic light to heavy ratios. Light versus heavy isotopic probe-modified peptide ratios are calculated by taking the mean of the ratios of each replicate paired light versus heavy precursor abundance for all peptide-spectral matches associated with a peptide. The paired abundances were also used to calculate a paired sample *t* test P value in an effort to estimate constancy in paired abundances and significance in change between treatment and control. P values were corrected using the Benjamini–Hochberg method.

### Gel-Based ABPP

Recombinant DCAF16 (MyBioSource.com, MBS1375983) (0.1 μg/sample) was pretreated with either DMSO vehicle or covalent ligand at 37 °C for 30 min in 25 μL of PBS, and subsequently treated with of IA-Rhodamine (concentrations designated in figure legends) (Setareh Biotech) at room temperature for 1 h in the dark. The reaction was stopped by addition of 4 × reducing Laemmli SDS sample loading buffer (Alfa Aesar). After boiling at 95 °C for 5 min, the samples were separated on precast 4–20% Criterion TGX gels (Bio-Rad). Probe-labeled proteins were analyzed by in-gel fluorescence using a ChemiDoc MP (Bio-Rad). Imaged gels were stained using Pierce Silver Stain Kit (Thermo Scientific, 24612) following manufacturer’s instructions.

### ML1–50-Competed Targets from ML2–33 Probe Pulldown Proteomics

HEK293T cells were harvested, lysed, and the proteome concentration was adjusted to 5 mg/mL in 500 μL of PBS using the BCA assay. HEK293T cell lysate were pretreated with DMSO vehicle or ML1–50 (200 μM) for 1 h at room temperature prior to ML2–33 probe labeling (20 μM) at room temperature for 1 h. To each tube containing cell lysate, the following reagents were added: 10 μL of 10 mM biotin picolyl azide (Sigma-Aldrich, 900912) in DMSO, 10 μL of 50 mM TCEP in H_2_O, 10 μL of 50 mM CuSO_4_ in H_2_O, and 30 μL of TBTA ligand (1.7 mM in 1:4 DMSO/tBuOH, Cayman Chemical, 18816). The reaction mixture was incubated at room temperature for 60 min, and the reaction was quenched by protein precipitation. Precipitated pellets were washed using 500 μL of MeOH and centrifuged again to yield white pellets. Samples were resuspended in 1.2% SDS-PBS (1 mL), completely dissolved, and heated to 90 °C for 5 min. The soluble proteome was then diluted with 5 mL of PBS and further incubated with high-capacity streptavidin-agarose beads (100 μL/sample, ThermoFisher Scientific, 20357). Beads and lysates were incubated overnight at 4 °C with rotation. On the following day, beads were suspended and washed three times with 0.1% SDS-PBS, PBS, and H_2_O. Washed beads were resuspended in 6 M Urea/PBS (500 μL), and the samples were further treated with DTT and iodoacetamide. After removing the supernatant, beads were resuspended in 100 μL of 50 mM TEAB and enzymatically digested overnight using sequencing-grade trypsin (Promega, V5111). Digested peptides were eluted through centrifugation and labeled using commercially available TMTsixplex tags (ThermoFisher, P/N 90061). After labeling, 35 μg of each labeled sample was combined and dried using a vacufuge. Dried samples were redissolved with 300 μL of 0.1% TFA in H_2_O and further fractionated using high-pH reversed-phase peptide fractionation kits (ThermoFisher, P/N 84868) following the manufacturer’s protocol. Dried fractions were then resuspended in 25 μL of 0.1% Formic acid/H_2_O (w/v) to be analyzed by LC-MS/MS.

### Mapping of ML1–50 Site of Modification on DCAF16 by LC-MS/MS

Pure DCAF16 protein (40 μg, MyBioSource.com, MBS1375983) was diluted in PBS (100 μL) and preincubated with ML1–50 (50 μM final concentration) for 30 min at room temperature. The protein was precipitated by the addition of 25 μL of TCA (100% w/v) and incubation at −80 °C overnight. The sample was then spun at 20,000g for 10 min at 4 °C. The supernatant was carefully removed, and the sample was washed three times with 200 μL of ice-cold 0.01 M HCl/90% acetone solution, with spinning at 20,000 for 5 min at 4 °C between washes. The sample was then resuspended in 30 μL of 8 M urea in PBS and 30 μL of ProteaseMax surfactant (20 μg/mL in 100 mM ammonium bicarbonate, Promega, V2071) with vortexing. Ammonium bicarbonate (40 μL, 100 mM) was then added for a final volume of 100 μL. The sample was reduced with 10 μL of TCEP (10 mM final concentration) for 30 min at 60 °C and alkylated with 10 μL of iodoacetamide (12.5 mM final concentration) for 30 min at 37 °C. The sample was then diluted with 120 μL of PBS before 1.2 μL of ProteaseMax surfactant (0.1 mg mL–1 in 100 mM ammonium bicarbonate, Promega, V2071) and sequencing grade trypsin (10 μL, 0.5 mg mL–1 in 50 mM ammonium bicarbonate, Promega, V5111) were added for overnight incubation at 37 °C. The next day, the sample was acidified with formic acid (5% final concentration) and fractionated using high pH reversed-phase peptide fractionation kits (Thermo Fisher, 84868) following manufacturer’s protocol.

### Quantitative TMT Proteomics Analysis

Cells were treated with either DMSO vehicle or compound (ML1–50 (1 μM, 24 h), ML1–71 (10 μM, 16 h), ML1–96 (10 μM, 16 h), ML2–5 (10 μM, 16 h), TH1–9 (5 μM, 16 h), ML2–9 (1 μM, 24 h)) and lysate was prepared as described above. Briefly, 25–100 μg protein from each sample was reduced, alkylated and tryptically digested overnight. Individual samples were then labeled with isobaric tags using commercially available TMTsixplex (Thermo Fisher Scientific, P/N 90061) kits, in accordance with the manufacturer’s protocols. Tagged samples (20 μg per sample) were combined, dried using a vacuum concentrator at 30 °C, resuspended with 300 μL 0.1% TFA in H_2_O, and fractionated using high pH reversed-phase peptide fractionation kits (Thermo Fisher Scientific, P/N 84868) according to the manufacturer’s protocol. Fractions were dried using a vacuum concentrator at 30 °C, resuspended with 50 μL 0.1% FA in H_2_O, and analyzed by LC-MS/MS as described below.

Quantitative TMT-based proteomic analysis was performed as previously described using a Thermo Eclipse with FAIMS LC-MS/MS.^5^ Acquired MS data was processed using ProLuCID search methodology in IP2 v.3-v.5 (Integrated Proteomics Applications, Inc.).^37^ Trypsin cleavage specificity (cleavage at K, R except if followed by P) allowed for up to 2 missed cleavages. Carbamidomethylation of cysteine was set as a fixed modification, methionine oxidation, and TMT-modification of N-termini and lysine residues were set as variable modifications. Reporter ion ratio calculations were performed using summed abundances with the most confident centroid selected from the 20 ppm window. Only peptide-to-spectrum matches that are unique assignments to a given identified protein within the total data set are considered for protein quantitation. High confidence protein identifications were reported with a < 1% false discovery rate (FDR) cutoff. Differential abundance significance was estimated using ANOVA with Benjamini-Hochberg correction to determine p-values.

### Knock Out Cell Line Generation

To generate a RNF126 knockout pool in HEK293T, we introduced Cas9 ribonucleoproteins (RNPs) complexed with a custom Alt-R sgRNA synthesized by IDT targeting exon 2 of the RNF126 genomic locus (guide sequence ATGCGAGTCTGGTTTTATCG). spCas9 and sgRNA were introduced into cells by nucleofection. Briefly, 1.6 μL of 62.5 μM Cas9 (IDT, #1081058), 2.88 μL of 50 μM sgRNA (Alt-R from IDT), and 0.52 μL of 1X phosphate-buffered saline were mixed and the RNPs were incubated at room temperature for 30 min. Subsequently, the RNPs were added to 200,000 HEK293T cells resuspended in 16.4 μL Nucleofector solution SF plus 3.6 μL of supplement. To this suspension, 1.2 μL of 100 μM Alt-R electroporation enhancer (IDT, #1075916) and 4.32 μL H_2_O were added for a final volume of 30 μL. This nucleofection mix was electroporated using a 4D Nucleofector X Unit with program DG-130 in a nucleofector strip. After 10 min of recovery, nucleofected cells were grown in a 6-well dish for 7 days. This RNF126 knockout pool was expanded and aliquoted for storage.

To isolate isogenic RNF126 knockout clones, the knockout pool was subjected to single cell sorting into 96-well plates using a WOLF microfluidic cell sorter (Nanocellect). Single cells were allowed to grow into colonies for 2 weeks, expanded further, and frozen into aliquots for storage. During cell expansion a sample of each clone was processed into lysate and RNF126 knockout clones were identified by anti-RNF126 immunoblotting (ProteinTech, #66647–1-Ig). Clone 2B8 was designated as the RNF126 knockout.

The DCAF16 knockout cell line was purchased from Ubigene with guide sequences AGAGGGGGCCATTCAGGAAT TGG and TTCTGACAAGTGGTCAGGAG AGG (catalog number YKO-H721).

### Site-Directed Mutagenesis on FLAG-Tagged DCAF16 Plasmid

Site-directed mutagenesis was performed on FLAG-tagged wild type DCAF16 plasmid (Origene, RC208716L3) using Q5 Site-Directed Mutagenesis Kit (NEB, E0552S) according to the manufacturer’s protocol. The sequences of the primers used are shown below.

C58S Primer (Forward): GCAGGTTAAGAGCCTTTTAAAATATTC.

C58S Primer (Reverse): CAGGCAAGACTCTCAAG.

C119S Primer (Forward): TCTGGCCTCTAGCGGAGTCCCAC.

C119S Primer (Reverse): GGGGGCCATTCAGGAATT.

### Plasmid Isolation

*E. coli* containing desired plasmids were pelleted, lysed, and neutralized using QIAGEN Plasmid Plus Midi Kit (Qiagen, 12943) according to the manufacturer’s protocol. The eluted plasmid concentrations were determined using Nanodrop quantification.

### Expression of FLAG-Tagged Wild Type DCAF16 and Mutants in DCAF16 Knockout Cells

For lentivirus production, FLAG-tagged wild type DCAF16 or FLAG-tagged DCAF16 mutant plasmids, pMD2.G (Addgene, 12259) and psPAX2 (Addgene, 12260) were transfected into HEK293T cells using Lipofectamine 2000 (ThermoFisher, 11668027). The virus-containing medium was collected and filtered after 48 h and was used to infect HEK293 DCAF16 knockout cells with 1:1000 dilution of Polybrene (Sigma-Adrich, TR-1003-G). After 48 h, the infected cells were selected with puromycin (2 μg/mL).

### DCAF16 Knockdown Studies

MISSION shRNA lentiviral construct, pMD2.G (Addgene, 12259) and psPAX2 (Addgene, 12260) were transfected into HEK293T cells using Lipofectamine 2000 (ThermoFisher, 11668027). The virus-containing medium was collected and filtered after 48 h and was used to infect target cells (K562, Mino or LNCaP cells) with 1:1000 dilution of Polybrene (Sigma-Adrich, TR-1003-G). After 48 h, the infected cells were selected with puromycin. MISSION pLKO.1-puro Non-Mammalian shRNA Control (Sigma-Adrich, SHC016) was used as a control shRNA.The shRNA sequence used for generation of DCAF16 knockdown lines is shown below.

shDCAF16 (Sigma-Aldrich, TRCN0000143155): CTCTAAATGGAGCACTGCAAT.

### RT-qPCR Analysis

Total RNA was extracted from cells using Monarch Total RNA Miniprep Kit (NEB, T2010S) according to the manufacturer’s protocol. cDNA was synthesized and gene expression was confirmed by qPCR using Luna Universal One-Step RT-qPCR Kit (NEB, E3005S) following the manufacturer’s protocol with the CFX Connect Real-Time PCR Detection System (BioRad). Relative DCAF16 gene expression was normalized to the GAPDH gene. The sequences of the qPCR primers are shown below.

GAPDH Primer (Forward): GTCTCCTCTGACTTCAACAGCG.

GAPDH Primer (Reverse): ACCACCCTGTTGCTGTAGC.

DCAF16 Primer #1 (Forward): TGACCACTTGTCAGAATCAGAA.

DCAF16 Primer #1 (Reverse): AGAGGCGATAAGTTGGGCAC.

DCAF16 Primer #2 (Forward): TGGATCCAAGCACACCAGTC.

DCAF16 Primer #2 (Reverse): TGGTTCCAGTTTGGGGACAC.

DCAF16 Primer #3 (Forward): CAATTCCTGAATGGCCCCCT.

DCAF16 Primer #3 (Reverse): GTGCTCCATTTAGAGTGGCA.

DCAF16 Primer #4 (Forward): AGTCTTGCCTGGCAGGTTAAG.

DCAF16 Primer #4 (Reverse): GGGACTTGTAAGAGGCTTTTGAA.

## Data Availability

The data sets generated during and/or analyzed during the current study are available from the corresponding author on reasonable request. We would be happy also provide the raw Western blotting data upon request. Data processing and statistical analysis algorithms from our lab can be found on our lab’s Github site: https://github.com/NomuraRG, and we can make any further code from this study available at reasonable request.

## Supporting Information Available

Supporting Table legends; Figure S1 on characterization of ML 1–50; Figure S2 on ML 1–71 effects on cell viability; Figure S3 on characterization of SMARCA2 degrader; Figure S4 on characterization of AR and BCR-ABL/c-ABL degraders; Figure S5 on characterization of BTK degraders Synthetic methods and characterization (PDF)
Table S1 on proteomics of ML 1–50 (XLSX)
Table S2 on chemoproteomic profiling of ML 1–50 (XLSX)
Table S3 on cysteine chemoproteomic profiling of ML 1–50 (XLSX)
Table S4 on proteomic profiling of DCAF16 KO cells (XLSX)
Table S5 on proteomic profiling of ML 1–71 (XLSX)
Table S6 on proteomic profiling of ML 2–9 (XLSX)
Table S7 on proteomic profiling of TH 1–9 (XLSX)
